# Sensory Perception in Bacterial Cyclic Diguanylate Signal Transduction

**DOI:** 10.1128/jb.00433-21

**Published:** 2022-02-15

**Authors:** Trevor E. Randall, Kelly Eckartt, Sravya Kakumanu, Alexa Price-Whelan, Lars E. P. Dietrich, Joe J. Harrison

**Affiliations:** a Department of Biological Sciences, University of Calgary, Calgary, Alberta, Canada; b Department of Biological Sciences, Columbia University, New York, New York, USA; Geisel School of Medicine at Dartmouth

**Keywords:** cyclic diguanylate, biofilms, sensor domain, signal transduction, stimulus perception, diguanylate cyclase, phosphodiesterase

## Abstract

Cyclic diguanylate (c-di-GMP) signal transduction systems provide bacteria with the ability to sense changing cell status or environmental conditions and then execute suitable physiological and social behaviors in response. In this review, we provide a comprehensive census of the stimuli and receptors that are linked to the modulation of intracellular c-di-GMP. Emerging evidence indicates that c-di-GMP networks sense light, surfaces, energy, redox potential, respiratory electron acceptors, temperature, and structurally diverse biotic and abiotic chemicals. Bioinformatic analysis of sensory domains in diguanylate cyclases and c-di-GMP-specific phosphodiesterases as well as the receptor complexes associated with them reveals that these functions are linked to a diverse repertoire of protein domain families. We describe the principles of stimulus perception learned from studying these modular sensory devices, illustrate how they are assembled in varied combinations with output domains, and summarize a system for classifying these sensor proteins based on their complexity. Biological information processing via c-di-GMP signal transduction not only is fundamental to bacterial survival in dynamic environments but also is being used to engineer gene expression circuitry and synthetic proteins with à la carte biochemical functionalities.

## INTRODUCTION

## BACTERIA USE c-di-GMP NETWORKS TO MAKE SENSE OF THEMSELVES AND THE WORLD

The ability of bacteria to monitor their cell status and surroundings is required for their survival in dynamic environments (1–3). Bacteria use a repertoire of signal transduction systems for these surveillance activities, including cyclic diguanylate [bis-(3′,5′)-cyclic diguanylate monophosphate, or c-di-GMP] networks. Because c-di-GMP signaling proteins regulate a variety of bacterial processes in phylogenetically distant species from dissimilar habitats, it is unsurprising that c-di-GMP networks sense remarkably diverse physical, chemical, and mechanical stimuli. These inputs include, for example, ambient light variations (4), thermal fluxes (5), cellular nucleotides (6), surfaces (7, 8), quorum-sensing (QS) molecules (9–12), and the toxic oxidants of the innate immune system (13), among many others.

Here, we use the term sensory perception to refer to the mechanisms responsible for converting an input stimulus into a biochemical output and the term stimulus to refer to any agent or change inside or outside the cell. Catalytic mechanisms for c-di-GMP synthesis and degradation are understood in detail (14–16), and the downstream processes leading to cellular responses via c-di-GMP-binding effectors have been covered in other excellent reviews (17–20). However, sensory perception by c-di-GMP signaling networks remains a knowledge gap. In this review, we contextualize information from bioinformatics resources and summarize a growing body of literature advancing an understanding of sensory perception in c-di-GMP signal transduction. This evaluation uncovers the complex sensory inputs found in c-di-GMP networks and reveals a nascent mechanistic understanding of how they work. The information compiled here will be a resource for bacteriologists seeking to advance the study of these networks.

## PRINCIPLES OF SENSORY PERCEPTION IN c-di-GMP SIGNALING

**FIG 1:**
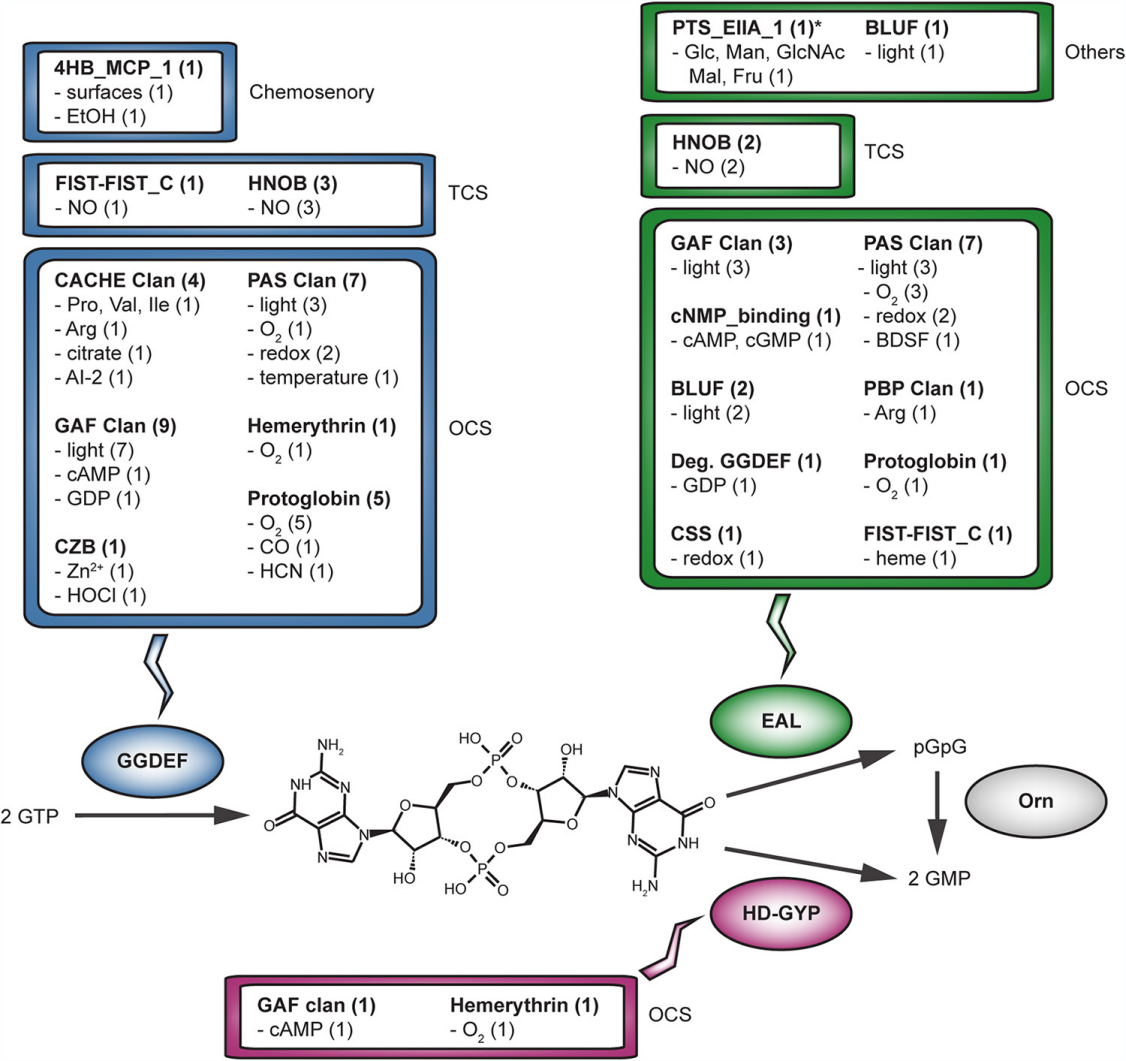
An overview of sensory domains found in c-di-GMP signaling proteins and their functions that have been substantiated *in vitro* and *in vivo*. Based on an analysis of 50 unique proteins from diverse bacterial species, integers in parentheses denote the number of unique proteins containing the sensory domain with the indicated function. All examples of sensor domains found in diguanylate cyclases or phosphodiesterases as well as their oligomeric receptor complexes are described in the text. Sensor domains have been categorized according to the complexity of the system in which they are found, namely, one-component system (OCS), two-component system (TCS), chemosensory system, or others.

c-di-GMP is synthesized by diguanylate cyclases (DGCs) that possess a GGDEF domain, and it is degraded by c-di-GMP-specific phosphodiesterases (PDEs) that possess an EAL or HD-GYP domain (18). Production of GMP from 5′-phosphoguanylyl-(3′,5′)-guanosine (pGpG), which is an intermediate c-di-GMP degradation product produced by EAL domain-containing PDEs, is carried out by the homeostatic functions of nanoribonucleases (21–23). DGCs are functionally active as protein dimers or higher order oligomers (8, 24), while EAL and HD-GYP domains are potentially functional as monomeric or oligomeric structures (25–28). All of these enzymes might respond to stimuli through mechanisms that modulate their transcription and translation. However, quick adjustments to c-di-GMP levels that drive just-in-time effector regulation might be best facilitated by posttranslational modification of DGCs and PDEs. In principle, the sensor and receiver domains found in these proteins and their oligomeric complexes enable fast, reversible activity modulation (Fig. 1).

We analyzed sensory inputs and domain architectures of proteins containing GGDEF, EAL, and/or HD-GYP domains using the Pfam database (see Table S1 in the supplemental material) (29), the National Center for Biotechnology Information (NCBI) Conserved Domain Database (CDD), and the NCBI Conserved Domain Architecture Retrieval Tool (CDART) (see Table S2 in the supplemental material) (30). A bioinformatic limitation is that many sensor domains remain unrecognized. However, some principles in our understanding of sensory inputs in c-di-GMP networks are emerging.

**FIG 2:**
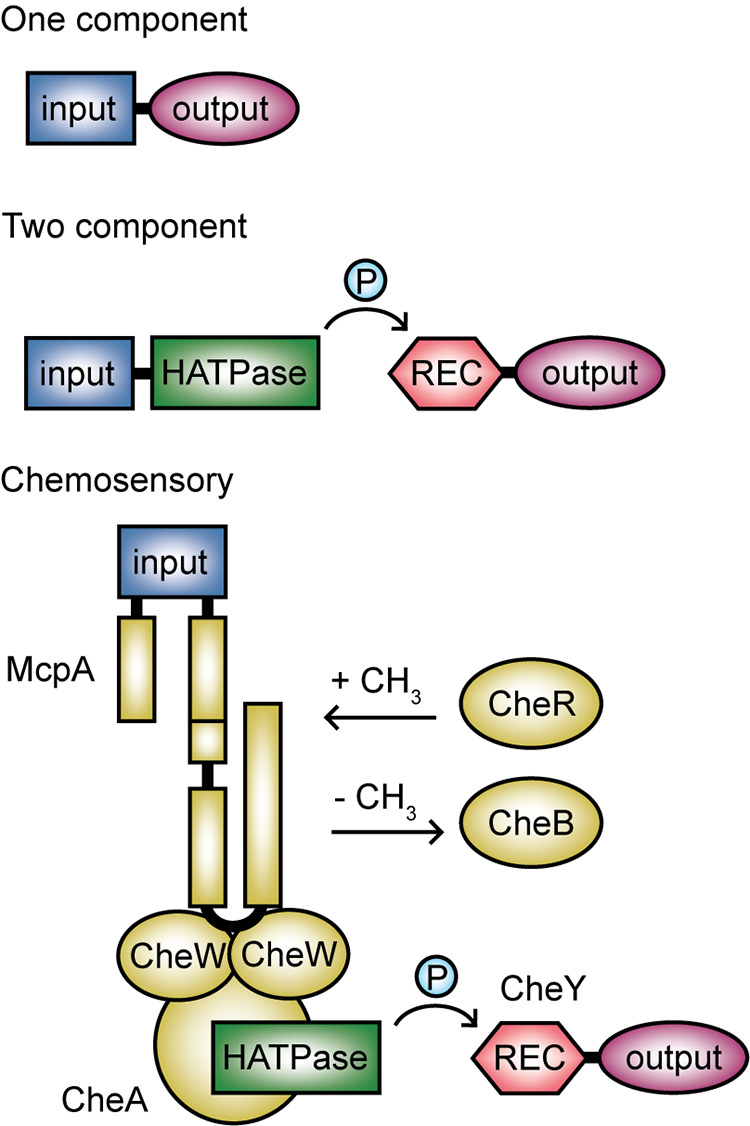
The complexity scheme provides a basis for classifying signal transduction proteins. One-component systems are comprised of 1 protein that codes for both input and output functions. Two-component systems are minimally comprised of 2 proteins, of which 1 includes a histidine kinase (HATPase) domain and 1 includes a phosphoreceiver (REC) domain that is phosphorylated by the kinase. Chemosensory systems are minimally comprised of 6 proteins with homology to the archetypal E. coli chemosensory complex, which also includes a histidine kinase and partner phosphoreceiver protein.

**FIG 3:**
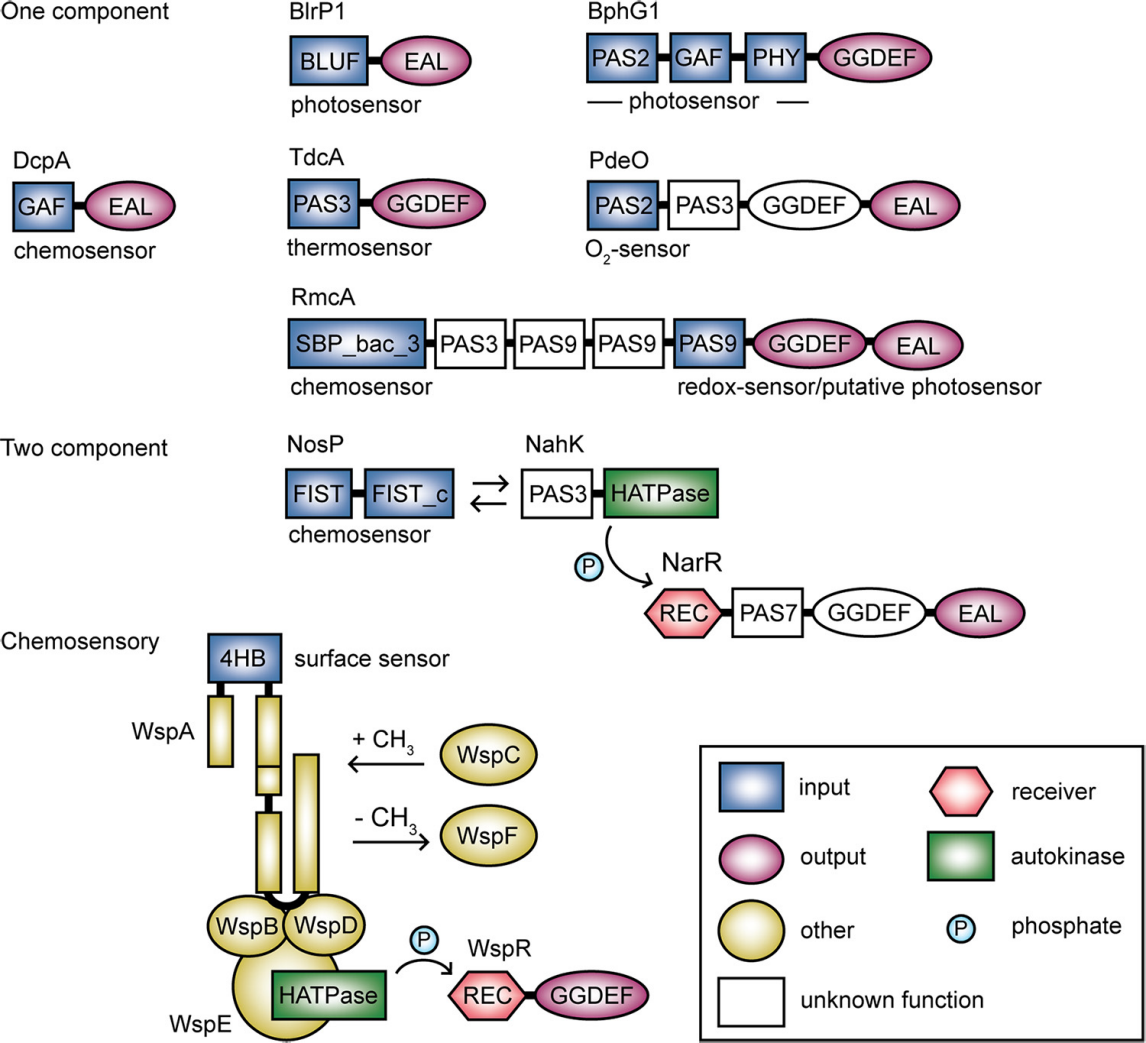
The complexity scheme classification illustrates the systematic but varied integration of sensory input domains in c-di-GMP signaling proteins and their receptor complexes. All examples of c-di-GMP signaling proteins are discussed in the main text. DGC and PDE proteins often function as higher order oligomers; however, for simplicity, protein oligomeric states are not illustrated here. Protein lengths are not drawn to scale. Transmembrane regions of proteins are not illustrated.

A key observation is that GGDEF, EAL, and HD-GYP domains are found in thousands of unique protein domain architectures with putative sensor and receiver domains (Tables S1 and S2). There are many ways of classifying signal transduction proteins. However, one practical system for grouping the diverse GGDEF, EAL, and HD-GYP domain-containing architectures is the complexity scheme (31–33), which classifies signal transduction proteins into one-component, two-component, and chemosensory systems (Fig. 2). While there are exceptions, most c-di-GMP signaling proteins discussed in this review can be classified by this scheme, which also provides knowledge about the integration of sensory inputs in c-di-GMP networks (Fig. 1 and 3).

One-component systems (OCSs) combine sensory and regulatory functions in a single protein (Fig. 2), although sensory functions can also be distributed between multiple domains. Also, some OCSs contain multiple sensory domains each with different functions. All of these protein configurations are relevant to c-di-GMP signaling (Fig. 3). OCSs are thought to comprise the predominant mode of sensing in prokaryotes and are hypothesized to be primarily intracellular, although a key subset of these proteins in c-di-GMP signaling contains periplasmic sensory domains (31, 34, 35).

In contrast, two-component systems (TCSs), which are mostly involved in sensing extracellular stimuli, are minimally comprised of a sensor histidine kinase and a cognate response regulator (Fig. 2). Some TCSs contain auxiliary components important for stimulus perception, which also have been observed in c-di-GMP networks and are key players in stimulus sensing (Fig. 3).

Lastly, chemosensory systems respond to extracellular stimuli and are the most complex. They are minimally comprised of 6 proteins that are homologous to the Escherichia coli McpA, CheA, CheW, CheR, CheB, and CheY proteins, which correspond to the methylating chemotaxis protein, histidine kinase, coupling protein, methyltransferase, methylesterase, and response regulator of the chemosensory pathways regulating flagellar motility, respectively (Fig. 2) (33, 36). However, while there are 19 recognized classes of chemosensory systems, those modulating c-di-GMP fall into 2 classes that are either associated with alternative cellular functions (class ACF) (Fig. 3) or type IV pilus (T4P) motility (class TFP) (36). Taken together, the complexity scheme classification illustrates that modular inputs are integrated into c-di-GMP signaling proteins and their associated receptor complexes in systematic but intricately varied ways.

## MODULAR SENSORY AND RECEIVER DOMAINS FOUND IN DGCs AND c-di-GMP-SPECIFIC PDEs

**FIG 4:**
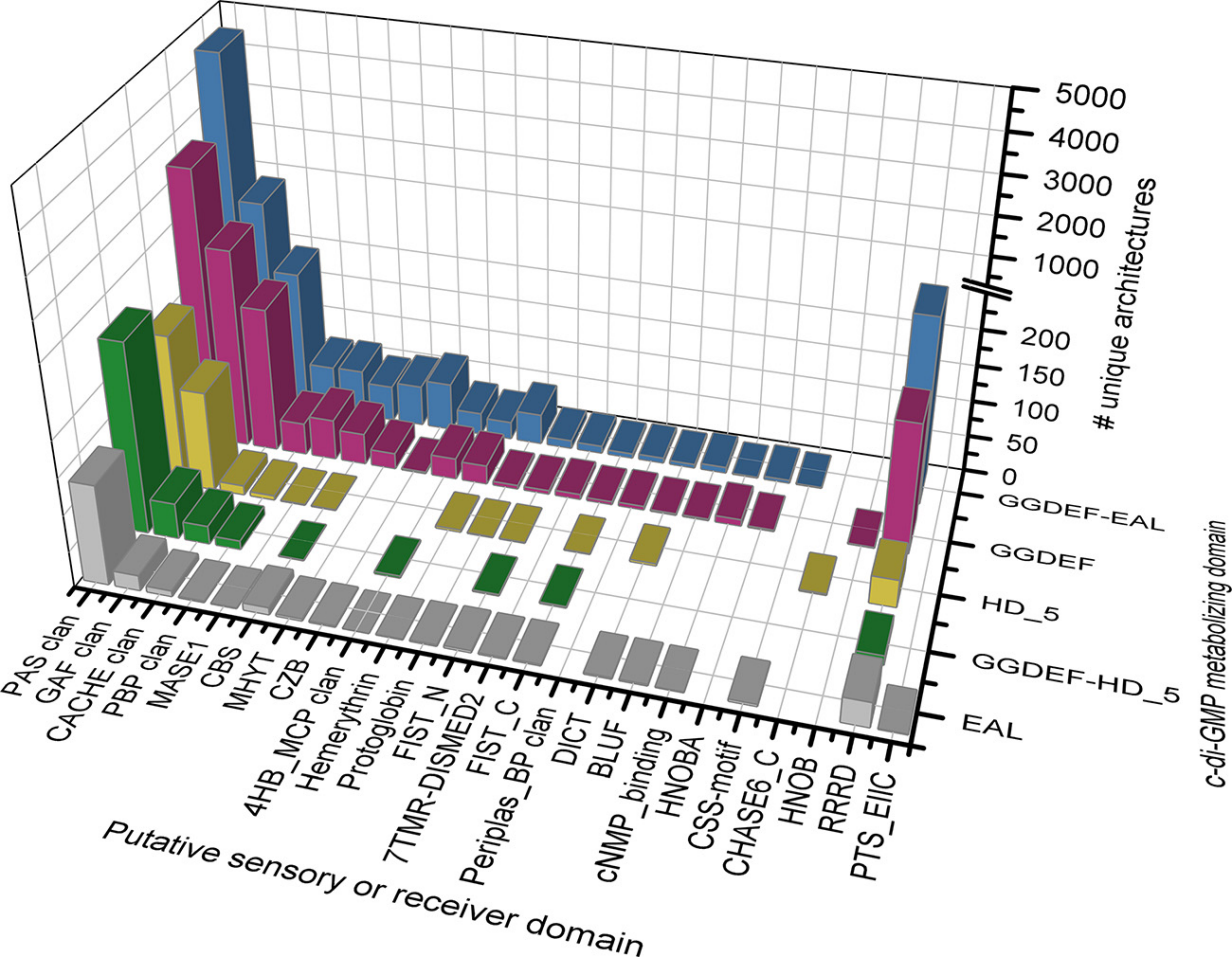
Identity, abundance, and diversity of putative sensory and receiver domains found in the conserved domain architectures of c-di-GMP signaling proteins. Counts represent the number of unique protein domain architectures that contain 1 or more of the indicated putative sensory or receiver domains and a putative GGDEF, EAL, and/or HD_5 domain. Pfam clans contain >1 domain family, and architectures were counted more than once in the clan categories if they contain >1 identifiable domain family from that clan. The Pfam HD_5 domain encompasses many but not all the architectures containing the CDD HD-GYP domain. Putative sensory domains are ordered by their overall abundance in DGCs and PDEs; the 2 representative receiver domains (RRRD and PTS_EIIC) are positioned on the right side of the *x* axis. Domain architectures were retrieved from the Pfam database v34.0 on 24 July 2021. This list is nonexhaustive and highlights domains discussed in the text. See Table S1 for additional data and accession numbers.

Bioinformatics is useful for identifying sensory domains in c-di-GMP signaling proteins even if the technology does not yet make it possible for all domains to be identified. For example, the Pfam database is structured into domain clans and domain families. Domain clans are comprised of unique domain families thought to have arisen from a single evolutionary origin (see the work of Finn and colleagues [37] for the bioinformatic criteria used in these groupings). Pfam recognizes dozens of putative sensor protein domain families that are linked to unique conserved domain architectures containing GGDEF, EAL, and/or HD_5 domains (Fig. 4; Table S1), of which the HD_5 domain encompasses many but not all architectures containing the CDD HD-GYP domain. Comparable results can also be discerned using the CDD classification and CDART (Table S2). These analyses predict diverse input functions among DGCs and c-di-GMP-specific PDEs. Although it must be acknowledged that there are biases in genomes and annotation coverage across the bacterial tree of life (38) and key differences between databases (Tables S1 and S2), some sensory domains—such as those belonging to the Per-Arnt-SIM (PAS); cGMP-phosphodiesterase, *Anabaena* adenylyl cyclases, and E. coli FhlA (GAF); and calcium channels and chemotaxis receptors (CACHE) domain clans—are evidently prevalent among these unique conserved architectures (Fig. 4; Tables S1 and S2).

In the sections that follow, we highlight some of the sensor and receiver domains that are found most frequently in combination with GGDEF, EAL, and HD-GYP domains and their complexes as well as those that are best studied even if they are relatively rare. The interpretation of protein domain bioinformatics can be daunting for microbiologists because (i) different databases define protein domains using algorithms that group similar domains differently, (ii) different algorithms are varied in their ability to detect protein domains, and (iii) nomenclature can vary between databases and is not always consistent with relationships deduced from sequence alignments or statistical models. We highlight the relevant differences in nomenclature between databases, as well as the trends and knowledge gaps in c-di-GMP signaling that emerge from this assessment. We also summarize basic principles from biochemistry explaining how these modular input devices regulate the activity of output domains. In the sections that follow, sensory domains with known functions are presented first and in their order of overall abundance in GDDEF, EAL, and HD_5 (HD-GYP)-domain containing proteins (Table S1); putative phosphoreceiver domains are discussed afterward. Regardless of their abundance, putative sensory domains with unknown functions are discussed together in a final section.

### The Per-Arnt-SIM (PAS) domain clan.

PAS domains are cytoplasmic sensory domains found in all kingdoms of life (39, 40). PAS is by far the most frequently identified sensory domain found in association with GGDEF, EAL, and HD-GYP domains (Fig. 4; Tables S1 and S2). PAS domains are highly diverse. Pfam v34.0 subdivides the PAS domain clan into 17 families, and it also includes the Light, Oxygen, Voltage (LOV) domain, which is a part of the PAS9 family. PAS domains are minimally dimeric and are ∼100 amino acids long (41). They are characterized by the PAS fold, which is comprised of a core 5-stranded antiparallel β-sheet that has several flanking α-helices (42, 43); the flanking α-helices are often involved in oligomerization (44, 45). Despite the conserved three-dimensional structure of the PAS fold, PAS domains have primary sequence similarities as low as 20% (43). Divergence in PAS domains has resulted in varied sensory functions arising from differences in substrate and cofactor binding. In c-di-GMP signaling, for example, PAS domains mediate light and redox sensing via flavin cofactors (46–49) and oxygen sensing via heme groups (50, 51). In contrast, the temperature-sensing, thermosensitive Per-Arnt-SIM (thermoPAS) domain found in DGCs does not appear to have any cofactor at all (5). Sensory perception by PAS domains likely proceeds by a shared mechanism; stimuli (e.g., photons and O_2_) elicit rearrangements in the core PAS fold β-sheet structure that are propagated to the output domain via torque of a linker region situated at the C terminus of the PAS domain (41, 43). This structural alteration initiates signal transduction by affecting the catalytic sites in the output domain so that its activity is changed and/or promotes a change in oligomerization state (e.g., by exposing a protein-binding interface) that affects protein activity (41, 43).

### The cGMP-phosphodiesterase, *Anabaena* adenylyl cyclases, and E. coli FhlA (GAF) domain.

The GAF domain is a cytoplasmic sensory domain (52). The Pfam v34.0 GAF clan is subdivided into 14 different families and includes GAF domains found in phytochromes and cyanobacteriochromes (part of the GAF1 family), which have been studied for their roles in c-di-GMP-dependent light sensing (53, 54). However, we note that in CDART, the three-domain arrangement found in phytochromes (PAS2-GAF-PHY, described in the section on photosensing) is denoted COG4251 (Table S2). The GAF domain is related to the PAS domain (55, 56); however, the primary GAF fold contains a 6-stranded antiparallel β-sheet with 2 to 4 stabilizing α-helices (43, 55). Sensory perception by GAF domains originates from stimulus-induced conformational changes within the binding pocket of the antiparallel β-sheet, generating torsion that leads to conformational changes in the rest of the domain (55, 57). Structural changes are presumably propagated through a linker to the adjacent output domain.

### The calcium channels and chemotaxis receptors (CACHE)-like domain clan.

The CACHE-like domain clan is thought to be a group of primarily cell surface receptors and might be the most abundant group of extracellular sensory modules in prokaryotes (58). The CACHE-like clan is currently divided into 25 families in Pfam v34.0, including the dCache_1, sCache_3_3, CHASE1, CHASE4, CHASE8, GAPES1, and YkuI_C domains, which are all found in DGCs and c-di-GMP-specific PDEs. We note that the YkuI_C domain is one of 2 small families of CACHE-like domains that is thought to be intracellular (58). Structurally, CACHE domains are predicted to have N- and C-terminal transmembrane (TM) α-helices (59), with three strands in between them that form a β-sheet that is similar to the PAS fold (58). The CACHE-like domains are thought to propagate signals via the C-terminal TM helix to an intracellular output domain (58). One proposed mechanism for TM signal transduction is the piston model (60) in which conformational changes in the periplasmic CACHE domain push the C-terminal TM helix through the membrane, activating the adjacent output domain (61).

### The periplasmic binding protein (PBP) clan.

The PBP clan is prevalent in proteins with GGDEF, EAL, and/or HD-GYP domains (Fig. 4; Tables S1 and S2), and yet among its 27 member families in the Pfam v34.0 database, only the SBP_bac_3 domain has been studied in c-di-GMP networks (it is found in the Pseudomonas aeruginosa RmcA protein and functions in l-arginine sensing [34] [Fig. 3]). Domains of the PBP clan are periplasmic and may bind diverse biomolecules, including amino acids, sugars, metal ions, and peptides (62, 63). In the literature, PBP clan domains are sometimes called Venus flytrap (VFT) or Pacman domains (64) because they consist of two large lobes that close around a bound ligand (65–67). The two lobes are connected by a 2-stranded antiparallel β-sheet hinge region (67, 68) that forms a deep cleft for ligand binding (65, 67, 68). Ligands become trapped in the cleft and the subsequent large conformational changes are presumably transferred to a cytoplasmic output domain through a TM linker (34, 64).

### The chemoreceptor zinc-binding (CZB) domain.

The CZB domain is a zinc (Zn)-binding domain that facilitates hypochlorite and/or Zn sensing in some DGCs (69, 70). The CZB domain contains an antiparallel α-helical bundle that functions as part of a symmetric homodimer (69). Zn^2+^ coordination is facilitated by 3 histidines and 1 cysteine that are conserved among CZB domains. Although the mechanism of signal propagation is not known, it is hypothesized that metal binding causes a conformational change in the α-helical bundle that is transmitted to the output domain through a linker (69, 70).

### The hemerythrin domain.

Named for invertebrate hemerythrins, which are O_2_-carrier proteins, hemerythrin domains are sometimes denoted bacterial hemerythrin-like (Bhr), particularly in the context of c-di-GMP signaling. Hemerythrin domains are defined by a binuclear nonheme iron center in which 7 amino acids coordinate 2 iron (Fe) ions (71). The binding of O_2_ to this di-iron center causes it to oxidize from the diferrous (Fe^2+^) to diferric (Fe^3+^) state (71, 72). While the impact of this changed oxidation state on protein structure is unknown, evidence indicates that it controls the activity of the adjacent output domain (71, 73).

### The protoglobin domain and the globin-coupled sensor (GCS).

The chimeric globins, which belong to the CDD globin-like domain family, include the prokaryotic globin-coupled sensors (GCSs) (74) that have been identified in several DGCs and PDEs (75). The Pfam v34.0 database identifies many of these domains as protoglobins (Fig. 4 and Table S1). The GCSs have the canonical 3-over-3 α-helical globin fold, with Fe(II)-heme covalently bound to a histidine amino acid in a binding pocket formed by 4 of the α-helices (75–77). In c-di-GMP signaling, the GCSs function in O_2_ sensing. Based on *in vitro* reactivity, however, it is possible that GCSs could function in CO (76, 78, 79) or HCN sensing (76, 78, 79), although a physiological role for these latter functions has not been established. Coordination of O_2_ in the binding pocket by Fe(II)-heme and distal threonine and tyrosine residues (80) causes the α-helices to rearrange (75, 76, 78, 79, 81), and this change is transmitted to the adjacent output domain through a coiled-coil region at the C terminus of the GCS (77).

### The heme-nitric oxide-binding (HNOB) and HNOB-associated (HNOBA) domains.

The CDD and Pfam HNOB domain (82, 83) encompass many proteins that also belong to the Interpro H-NOX domain superfamily (84), which was first defined in the SONO protein found in *Clostridium* spp. (85). These domains are heme-based sensors that bind NO with femtomolar sensitivity (85). The HNOB domain consists of 7 α-helices and a 4-stranded antiparallel β-sheet in which heme is coordinated in a binding pocket (86). NO binding by heme in HNOB twists the cofactor in its binding pocket (87), altering the position of heme-coordinated histidine and proline amino acids and causing the outer loops of the domain to shift. This change presumably alters the function of the adjacent output domain (87, 88). In c-di-GMP signaling, our analysis suggests that HNOB and HNOBA might be associated only with proteins that have GGDEF domains (Fig. 3, Table S1 and S2); however, HNOB domains are found in auxiliary proteins of TCSs that modulate the activity of PDEs (83, 89–91).

### The blue-light using FAD (BLUF) domain.

The BLUF domain is well studied yet rare among GGDEF and EAL domain-containing proteins. It does not appear to be found in HD_5 (or HD-GYP) domain-containing proteins at all (Fig. 4; Tables S1 and S2). BLUF senses blue light using a flavin adenine dinucleotide (FAD) cofactor bound to fold that is characteristic of the BLUF family (92). The BLUF domain contains 4 α-helices and a 5-stranded antiparallel β-sheet, with 2 additional α-helices at the C terminus called the “helical cap” (46). Photon absorption induces structural changes in the BLUF domain that are propagated to an adjacent output domain to regulate it allosterically. A detailed mechanism of sensory perception by the BLUF domain-containing c-di-GMP-specific PDE BlrP1 (26, 46, 93–97) is discussed in the section on photosensing.

### The cyclic nucleotide-binding (cNMP_binding) domain.

cNMP_binding domains, encompassed within the CAP_ED domains in CDD (Table S2), are intracellular sensory domains that bind cGMP or cAMP (6, 98). First identified in DNA-binding factors (99) and nucleotide-gated ion channels (100), cNMP_binding domains are also associated with c-di-GMP-metabolizing enzymes (Fig. 4) (6). Structurally, this domain consists of an 8-stranded antiparallel β-sheet, an α-helical hinge region, and a C-terminal α-helical linker (99), with the helical linker promoting protein dimerization (6). Upon cNMP binding, the β-sheet swings inward, changing the conformation of the hinge domain, in turn altering the conformation of the linker, causing the activation of the associated output domain (6).

### The CSS motif domain.

This domain is always found at the N terminus of an EAL domain. Although its structure is unknown, the CSS motif domain is comprised of a periplasmic region flanked by putative TM helices (101). The periplasmic region contains 2 cysteine residues that form a disulfide bond under oxidizing conditions, which is thought to function like a redox-sensitive switch (101, 102). The CSS motif is discussed in detail in the section on O_2_ and redox sensing.

### The response regulator receiver domain (RRRD).

The Pfam RRRD, which is also termed the phosphoacceptor receiver (REC) domain by the CDD (103), is a phosphoreceiver that is posttranslationally modified by a partner kinase (104). Many GGDEF, EAL, and/or HD_5 (or HD-GYP) domain-containing proteins are predicted to have this domain (Fig. 4; Tables S1 and S2). This observation suggests that posttranslational modification via phosphorylation in TCSs or chemosensory systems frequently underpins sensory perception in c-di-GMP networks. Structurally, RRRD has a central 5-stranded parallel β-sheet that is situated in between 5 alternating α-helices (2 on one side of the parallel β-sheet, 3 on the other) (105). The central 3 β-strands contain 6 residues that are essential for phosphotransfer from the kinase to the target aspartate residue of the RRRD, which is phosphorylated (106–108). Within this phosphorylation pocket, the phosphoryl group ultimately forms several hydrogen bonds and salt bridges that drive positional changes in the C-terminal β-strands of the RRRD, changing the activity of the adjacent output domain (105).

### The phosphoenolpyruvate-dependent sugar phosphotransferase system EIIA 1 and EIIC (PTS_EIIA_1 and PTS_EIIC, respectively) domains.

Although rare in c-di-GMP signaling proteins (Fig. 4), the PTS_EIIA_1 and PTS_EIIC domains are phosphoreceiver domains (109) that otherwise comprise parts of the sugar phosphotransferase (PTS) system that was discovered in the 1960s (110). In the PTS system, enzyme I (EI) transfers the phosphoryl group of phosphoenolpyruvate to a variety of enzyme II (EII) proteins in response to specific sugars (110). EII subsequently acts to phosphorylate the sugar and aid in sugar import (110). The phosphorylation state of PTS_EIIA_1 and PTS_EIIC domains control the activity of an associated output domain or partner protein through interactions that mediate conformational changes (111).

### Other notable putative sensory domains with unknown functions.

The bacterial signaling protein N-terminal repeat (MHYT) domain, named after its conserved methionine-histidine-tyrosine-threonine amino acid pattern (112), is an integral membrane sensor domain. The MHYT domain structure is predicted to consist of 6 antiparallel TM α-helices connected by short cytoplasmic and periplasmic loops rich in charged amino acids (112). The activity of the MHYT domain is unknown; however, it appears relatively common among proteins with GGDEF and EAL domains (Fig. 4).

The FIST-FIST_C domain architecture is also present in some proteins containing GGDEF, EAL, and HD-GYP domains (Fig. 4). It has been observed in some auxiliary proteins of TCSs that regulate EAL domain-containing proteins (Fig. 3). Although evidence suggests that FIST-FIST_C may bind NO (113, 114) and/or heme (115), little is known about the mechanism of substrate binding and signal propagation.

The Pfam v34.0 aspartate chemoreceptor signal-transduction ligand-binding clan (4HB_MCP) contains both the chemoreceptor 4HB_MCP_1 family (which overlaps with the Tar_Tsr_LBD domain from CDD) (116) and the CHASE3 family. The 4HB_MCP clan domains are predicted to have a conserved 4-stranded α-helical bundle (116, 117). The function of CHASE3 is unknown, and our salient knowledge of the 4HB_MCP_1 domain in c-di-GMP signaling is discussed later in the context of the Wsp surface-sensing apparatus (118, 119).

Many putative sensory domains that are associated with GGDEF, EAL, and HD-GYP domain-containing proteins (Fig. 4 and Table S1 and S2) do not have experimentally validated roles. However, some generic, homology-derived functional predictions can be made for a few. For example, 7 transmembrane receptors with diverse intracellular signaling modules extracellular domain 2 (7TMR-DISMED2) is a TM domain predicted to sense carbohydrates via a conserved jellyroll fold (120). The cystathionine beta-synthase (CBS) domains are thought to be energy-sensing modules that may bind AMP, ATP, and *S*-adenosylmethionine (121). The periplasmic binding protein-like (Periplas_BP) domain, which is also found in LacI family transcriptional regulators (122), may bind to diverse solutes, including sugars. Many other putative sensory domains have no discernible physiological purpose, including the domain associated with diguanylate cyclases and phosphodiesterases and two-component systems (DICT), the membrane-associated sensor 1 (e.g., MASE1), and the cyclases/histidine kinases associated sensory extracellular 6C (CHASE6_C) domain (117), which is only associated with HD_5 (or HD-GYP) domains (Fig. 4, Table S1 and S2).

## MAKING SENSE OF IT ALL: THE SENSORY FUNCTIONS OF c-di-GMP NETWORKS

Here, we categorize the sensory functions of c-di-GMP signaling networks, describe the physiological relevance of several signal transduction mechanisms that have been substantiated *in vitro* and *in vivo*, and highlight some of the important limitations and questions that remain.

The literature is rife with descriptions of genetic linkages between stimuli and DGCs or PDEs causing changes in intracellular c-di-GMP. While these observations are discussed and summarized in this review (see Table S3 in the supplemental material), we caution that mechanisms of sensory perception can be difficult to interpret from genetic linkages without additional data. For instance, deletion of a housekeeping PDE gene might have a dominant gain-of-function phenotype (elevated cellular c-di-GMP levels) that may confound interpretations of the genetic linkage. Similarly, a pitfall may come from deletion of a DGC gene that is highly expressed under the conditions tested, thereby producing a dominant loss-of-function phenotype.

Additionally, a small number of reports make putative stimuli-receptor linkages relying solely on c-di-GMP-dependent transcriptional reporter measurements as a proxy for direct, quantitative c-di-GMP measurements. Although c-di-GMP bioreporters are easy, quick, and inexpensive compared with more laborious analytical chemistry protocols relying on mass spectrometry infrastructure for quantifying c-di-GMP, reports relying solely on bioreporters have been excluded from this review because without direct measures of c-di-GMP levels, we cannot rule out that gene transcription might be controlled by other factors that are c-di-GMP -independent.

### Photosensing.

Light sensing is one of the best understood sensory functions of c-di-GMP networks. It is facilitated by photosensitive domains in (i) GGDEF and EAL domain-containing proteins or (ii) short photoreceptor proteins that modulate the activity of EAL domain-containing proteins through protein-protein interactions. Photoreceptors identified in c-di-GMP networks use light-sensitive BLUF, GAF, or LOV (PAS9) domains (Table 1). Compelling genetic linkages have been made between light-dependent bacterial behaviors and putative photoreceptors with c-di-GMP signaling functions in Pseudomonas aeruginosa (123), *Xanthomonas* spp. (124, 125), and Thermosynechococcus elongatus (4, 54, 126–128). Biochemical evidence for photosensitive catalysis has been demonstrated *in vitro* for DGCs and PDEs from many other species (Table 1), and in some cases, a detailed mechanism(s) of photoactivation is known.

**TABLE 1:** Photoreceptors in c-di-GMP networks

Bacterial species	Protein	Light^*a*^	Sensory domain, and/or cofactor	Function	Evidence^*b*^	Reference(s)
Synechococcus elongatus	SL2	Blue (+)	PAS9^*c*^, FMN	PDE	IDA	47
Pseudomonas aeruginosa	RmcA	Blue (+)	PAS9^*c*^, FAD	PDE	IMP	123
Klebsiella pneumoniae	BlrP1	Blue (+)	BLUF, FAD	PDE	IDA	26, 46, 93–95, 97, 219
Allochromatium vinosum	BldP	Blue (+)	BLUF, FAD	PDE	IMP, IDA	212
Magnetococcus marinus	BldP	Blue (+)	BLUF, FAD	PDE	IMP, IDA	212
Rhodopseudomonas palustris	PapB	Blue (+)	BLUF, FAD	regulator	IMP, IDA	96, 129
	PapA			PDE	IDA	
Thermosynechococcus elongatus	SesA	Blue (+), green^*d*^ (−)	GAF1, phycoviolobilin	DGC	IMP, IDA	4, 126, 127
	SesB	Blue (−), teal^*d*^ (+)	GAF1, phycoviolobilin	PDE	IMP, IDA	4, 54, 128
	SesC	Blue (+DGC, −PDE), green (+PDE, −DGC)^*d*^	GAF1, phycoviolobilin	DGC-PDE	IMP, IDA	4
*Synechocystis* sp. strain PCC 6803	Cip1	Far red^*d*^ (+)	GAF1, unknown	DGC	IMP, IDA	220
	Cph2	Far red^*d*^ (−)	GAF1-PHY, phycoviolobilin	DGC	IMP, IDA	131, 221
	Cph2	Blue (+), green (−)	GAF1, phycocyanobilin	DGC	IMP, IDA	131, 220, 221
*Idiomarina* sp. strain A28L	IsPadC	Red^*d*^ (+)	PAS2-GAF1-PHY, biliverdin IXα	DGC	IMP, IDA	54, 222–224
*Thioalkalivibrio* sp. strain ALMg3	TsPadC	Red^*d*^ (+)	PAS2-GAF1-PHY, biliverdin IXα	DGC	IMP, IDA	54, 224
Rhodobacter sphaeroides	BphG1	Far red^*d*^ (+)	PAS2-GAF1-PHY, biliverdin IXα	DGC	IDA	54, 225
Xanthomonas oryzae pv. Oryzae	XooBphP	Far red^*d*^ (+)	PAS2-GAF1-PHY, biliverdin IXα	PDE	IMP	124

a The (+) denotes an activator of the protein, whereas the (−) denotes an inhibitor.

b Categorized using gene ontology (GO) evidence codes, as follows: IDA, inferred from direct assay; IMP, inferred from mutant phenotype.

c The Pfam PAS9 includes the Light, Oxygen, and Voltage (LOV) domain, which is the sensor module found in this protein.

d Bilin chromophores found in cyanobacteriochromes and phytochromes have two reversible states with different absorption spectra; two colors have been included in instances where these two states require light of different frequencies for photoconversion (whereas the others may spontaneously relax to an unexcited state in the dark).

Perhaps the best studied photoreceptors in c-di-GMP signaling are PDEs that rely on the BLUF domain to sense blue light (Table 1). The archetype of these proteins is BlrP1 from Klebsiella pneumoniae, which consists of a BLUF domain linked to an EAL domain. Light increases the PDE activity of this protein 4-fold (26). A sophisticated mechanism of light sensing for BlrP1 was discerned in 2009 (26). Briefly, BlrP1 forms an antiparallel homodimer in which the EAL domains form a dimer interface comprised of 3 interacting helices from each of the BlrP1 monomers. Photon absorption by the FAD cofactor leads to a rearrangement of the hydrogen-bonding network between FAD and amino acids around the chromophore site within the BLUF domain. This structural change is propagated to the EAL domain of the opposing BlrP1 monomer through rearrangement of the β-strands and loop adjacent to the flavin, pushing the C-terminal helices of BLUF toward the dimer interface. Subsequently, this change applies force against the helices of the dimer interface, rotating the EAL domains with respect to each other, and repositioning the loop containing the amino acid residues that coordinate metal ions in the EAL active site (26, 95), which better exposes them to the substrate. Photon absorption, therefore, leads to allosteric activation of the EAL domain through changes in the quaternary structure of the BlrP1 dimer.

PapB from the purple nonsulfur bacterium Rhodopseudomonas palustris, which negatively regulates biofilm formation under blue light (129), is a short protein that contains a BLUF domain but no other identifiable protein domain. PapB interacts in a heterotetrameric complex with a partner protein, PapA, which contains a solo EAL domain (96). Simulation and site-directed mutagenesis (96, 129) suggest that light-induced conformational changes in PapB are propagated to the PapA EAL domain through the C-terminal α-helices of the PapB BLUF domain (130). Thus, by contrast to BlrP1, studies with PapB-PapA illustrate how EAL domain-containing proteins may interact with receptors in other proteins that modulate their activity as part of heteromeric complexes.

Another group of light-sensing DGCs and PDEs rely on GAF domains, of which all function as part of either phytochromes or cyanobacteriochromes (CBCRs) (Table 1). The GAF domains of both phytochromes and CBCRs possess a conserved cysteine residue that forms a stable covalent bond with a linear tetrapyrrole chromophore called a bilin (54). Examples of bilins found in these proteins include phycocyanobilin, biliverdin IXα, or phycoviolobilin (54) (Table 1). The phytochromes have a three-domain arrangement (PAS2-GAF1-PHY) that comprises the photosensory core module that incorporates the tetrapyrrole chromophore into its binding pocket (54), which is essential for the spectral properties of the proteins. In contrast, the CBCR variable domains may flank the GAF domain, and the photosensory core module is comprised of the GAF domain alone (54).

Phytochrome and CBCR proteins are regulated by a process called photoconversion (54). This mechanism presumably operates in DGCs and PDEs containing these photoreceptors too. In photoconversion, the bilin absorbs a photon of a wavelength corresponding to the effective length of its π-conjugated system (54), and in turn, the chromophore undergoes *Z/E* isomerization at a carbon double bond that causes the whole chromophore to rotate in its binding pocket (54, 131). This light-dependent “flip-and-rotate” mechanism (132) causes changes in hydrogen bonding and pi-pi interactions between the chromophore and its binding pocket (133), generating torsion that is transmitted via the C terminus of the phytochrome or CBCR to the adjacent output domain, initiating signal transduction activities.

Additionally, LOV domains, which are grouped in the Pfam PAS9 domain family with other structurally similar albeit functionally dissimilar domains, can regulate PDE activity. LOV domains bind flavin cofactors and undergo self-contained photocycles (134). In the absence of light (i.e., the inactive state), the flavin is noncovalently bound to the LOV domain. In the presence of light, (i.e., the active state), the flavin forms a covalent thioester bond to the protein via a conserved cysteine residue. Depending on the protein, the bond can reopen to the inactive state within minutes (47, 135). Light-sensing LOV domains have been studied in c-di-GMP-specific PDEs (Table 1), and here, we discuss a canonical one from the phototroph Synechococcus elongatus and a noncanonical one from the chemotroph P. aeruginosa.

The protein SL2, from the single-celled cyanobacterium *S. elongatus*, is a blue light-sensitive PDE that has been studied *in vitro*. SL2 contains a canonical LOV domain, a GGDEF domain, and an EAL domain. In the presence of blue light, a flavin mononucleotide (FMN) molecule in the LOV domain forms the canonical thioester bond between the flavin moiety and the key cysteine residue. The protein also creates electrostatic interactions between two arginine residues and the phosphate group of the FMN molecule (47). In this active state, the SL2 protein hydrolyzes c-di-GMP via its EAL domain (47).

In contrast, a PDE with a noncanonical LOV domain was recently implicated in an effect of light on biofilm development in the P. aeruginosa. In P. aeruginosa colony biofilms, high c-di-GMP levels stimulate the production of matrix polysaccharides that lead to the formation of vertical structures called “wrinkles.” Growth of these biofilms in the light leads to lower c-di-GMP levels and thereby inhibits wrinkling. The protein RmcA is a major PDE that contributes to the light-dependent inhibition of wrinkling (123). RmcA contains multiple sensory domains, including 4 PAS domains, and although it contains both a GGDEF domain and an EAL domain, genetic analyses indicate that c-di-GMP degradation is its primary activity *in vivo* (48). The deletion of the full RmcA protein resulted in a loss of inhibition of wrinkling when biofilms were grown in the light, meaning that Δ*rmcA* mutant biofilms wrinkled at the same time in both the light and the dark (123). Deleting just the fourth PAS domain (counting from the N terminus; “PASd”) of the RmcA protein was sufficient to elicit this phenotype, suggesting that this PAS domain functions to sense light and stimulate PDE activity (123). Prior work had indicated that RmcA binds a flavin molecule, raising the possibility that this cofactor could bind to the fourth PAS domain and that it could function as a light-sensing domain (48). Examination of the PASd amino acid sequence revealed that PASd does not harbor the characteristic cysteine residue which is thought to be necessary for light sensing in LOV domains (123). However, PASd does contain multiple other residues that are conserved in canonical LOV domains and shows overall homology to these domains. Furthermore, mutational studies of some LOV proteins have called into question the necessity of this cysteine residue for light sensing (135, 136). Taken together, these observations implicate RmcA-PASd and its effects on RmcA PDE activity in the light-dependent modulation of P. aeruginosa biofilm development via c-di-GMP signaling.

### Surface sensing (and mechanosensing).

Bacteria use mechanical sensing to respond to changes in fluid viscosity and surfaces; however, bacteria may also use chemical sensing for surface recognition. There are 3 apparatuses associated with surface recognition that have been genetically linked to c-di-GMP signal transduction (Table 2), as follows: (i) the Wsp apparatus (8, 118, 119, 137–139), (ii) the type IV pilus (e.g., the Pil-Chp system) (140, 141), and (iii) the flagellum (142–146). Many bacterial species possess >1 of these systems, and some, like P. aeruginosa, might possess all 3 (Table 2). However, many knowledge gaps remain in the mechanisms underlying c-di-GMP-dependent surface sensing for all of these systems.

**TABLE 2:** Examples of surface-sensing and/or mechanosensory apparatuses genetically linked to c-di-GMP signal transduction

Species	Sensory apparatus	Output DGC(s)/PDE(s)	Function(s)	Evidence^*a*^	Reference(s)
Caulobacter crescentus	Type IV pilus	PleD	DGC	IMP	149
	Flagellum stator	DgcB	DGC	IMP	145, 146
Pseudomonas aeruginosa	Wsp apparatus	WspR	DGC	IMP, IDA	8, 118, 119, 137–139
	Type IV pilus, Pil/Chp system	SadC	DGC	IMP	140
	Flagellum stator	SadC, SiaD	DGC, DGC	IMP, IEP	143, 144
Vibrio cholerae	Flagellum	CdgA, CdgL, CdgO	DGC, DGC, DGC	IMP, IEP	142

a Categorized using gene ontology (GO) evidence codes, as follows: IDA, inferred from direct assay; IEP, inferred from expression pattern; IMP, inferred from mutant phenotype.

The surface-sensing Wsp apparatus (Fig. 1) has been best studied in Pseudomonas species (8, 119, 137–139). This multicomponent apparatus is a type ACF chemosensory system (33, 36) that directly regulates the activity of the WspR DGC, which is activated by phosphorylation at a REC domain by the WspE histidine kinase (Fig. 3). Growth on surfaces activates the Wsp system (119, 137).

WspA, which is a transmembrane methylating chemotaxis protein, contains a putative periplasmic sensor 4HB_MCP_1 domain that is also present in other chemoreceptors (116). Surprisingly, chimeric WspA proteins in which the 4HB_MCP_1 domain is replaced with the ligand-binding domains from other P. aeruginosa chemotaxis receptors for amino acids (PctA, PctB, and PctC) or malate (CtpM) retain surface-sensing functions (119). This observation indicates that the periplasmic region of the receptor does not need to be conserved for the Wsp apparatus to respond to surfaces. Another study identified that ethanol increases c-di-GMP in P. aeruginosa and that this phenotype depends on *wspA* and *wspR* (7). Since ethanol taxis has been linked to numerous chemoreceptors with varied sensory domain types in the model bacterium Ralstonia pseudosolanacearum (147), it has been suggested that the TM region and signaling domain, which are common parts of these receptors, may be responsible for ethanol sensing (36, 147). Taken together, these observations have led to the hypothesis that the WspA TM regions are linked to receptor stimulation in response to surfaces. This interpretation is supported by observations that mutations in fatty acid biosynthesis pathways affecting membrane characteristics cause constitutive c-di-GMP production by the Wsp pathway (148). Nevertheless, the precise stimulus recognized by WspA and its sensory mechanism remain a mystery.

Type IV pilus (T4P)-dependent surface recognition has also been linked to c-di-GMP signaling in several bacterial species (Table 2) (149–153). Recent advances in chemical labeling of the T4P used in tandem with live-cell imaging have provided insight into potential mechanisms for T4P-mediated surface sensing (152), of which all have been presented in an elegant, recent review (152). Briefly, these putative mechanisms include force-induced pilin modifications, stimulation via the mechanosensitive von Willebrand Factor a (VWFa)-like domain of the pilus-tip protein, recognition of T4P subunits by TCSs, and/or obstruction of the T4P motor. All of these putative sensory mechanisms are incompletely understood; however, perhaps the best studied one with respect to c-di-GMP signaling is obstruction of T4P retraction in Caulobacter crescentus (149).

C. crescentus has tight adherence (tad) type IV pili and a single flagellum located at one of its cell poles. It uses these appendages to orchestrate the deployment of an exopolysaccharide holdfast within seconds of encountering a surface (154, 155). Production of the holdfast is regulated by c-di-GMP (156). Using cysteine-modified pilin subunits to facilitate thiol-reactive labeling of the T4P, Ellison and colleagues showed that cycles of pilus extension and retraction cease on surface contact and that this coincides with holdfast synthesis (149). Pilus retraction generates a measurable force. Physically blocking pilus retraction by chemically modifying the pilus with a bulky maleimide-polyethylene glycol molecule also stimulated holdfast production. Taken together, this result led the authors to conclude that T4P motor obstruction, rather than an attribute of the surface itself *per se*, stimulates surface recognition (149, 152). It was also observed that a mutant lacking *pleD*, which encodes a DGC, was stimulated for holdfast synthesis to a lesser extent than wild-type cells, implicating c-di-GMP in the surface-dependent stimulation of holdfast synthesis (149).

The flagellum is a target of c-di-GMP in many bacteria, and the outcome of this signaling is primarily inhibitory (157). For instance, c-di-GMP decreases the transcription of flagellar genes (158, 159), affects the translation of flagellar mRNAs (160), and allosterically modulates flagellar rotation and architecture (160, 161). However, recent findings indicate that the flagellum is also an activator of c-di-GMP synthesis (142, 157). This function has been linked to growth on surfaces (144, 145, 162), and accumulating evidence implicates the flagellar motor in a mechanosensitive, surface-dependent response (143–145). While the connections are not yet clear, these observations parallel findings demonstrating that the flagellum motor dynamically remodels in response to changes in physical load (163–165).

A c-di-GMP-dependent tactile response in which the flagellar motor acts as a sensor has also been best studied in C. crescentus (145). An understanding of flagellum-mediated mechanosensing has come from imaging this organism under flow in microfluidic devices, which contrasts to the static conditions used to dissect T4P obstruction in surface sensing. Under flow conditions, C. crescentus becomes tethered to a surface by its flagellum and then by its polar T4P. However, the ability of this bacterium to attach to a surface is increased in strains lacking outer parts of the flagellum (such as the rod, hook, and filament), suggesting that this organelle does not simply act as a tether (145, 166). The individual contributions of the flagellum and T4P to surface sensing were disentangled in narrow microfluidic chambers (the height of a single bacterium) in which pili are no longer required for surface attachment. In such chambers, flagellar motor rotation is essential for holdfast deployment (145). The DgcB diguanylate cyclase, which colocalizes with the flagellum at the cell pole, drives holdfast synthesis by producing c-di-GMP that allosterically activates the glycosyltransferase HfsJ. While the mechanism of DgcB activation is not yet understood *in vivo*, the tactile response might be propagated to the cytoplasm by a change in proton flux through the stators. This hypothesis is predicated on observations that DgcB activity is stimulated strongly by pH *in vitro* and that mutants lacking the MotB stator or expressing a MotB^D33N^ protein, which cannot conduct protons, fail to respond to surfaces (145).

### Oxygen and redox sensing.

In this section, we discuss key examples of proteins that link O_2_ and the cellular redox state to c-di-GMP signaling. O_2_-sensing proteins found in c-di-GMP networks often rely on a heme-binding protoglobin domain (e.g., the globin-coupled sensor) or PAS domain at the N terminus to regulate enzymatic activity in an O_2_-dependent manner (Table 3). Furthermore, a growing number of proteins have been identified that are sensitive to shifts in the redox potentials of cellular metabolites, such as those containing cysteine residues (with the potential to form disulfide bonds when oxidized, e.g., glutathione) (Table 3). There is overlap between these proteins and those that sense O_2_ because O_2_ is a strong oxidant that can react with cofactors that are also sensitive to cellular metabolites. To begin, we present examples of a DGC and PDE that sense O_2_ via protoglobin and PAS domains, respectively. Subsequently, we describe c-di-GMP-modulating proteins that sense redox via a hemerythrin domain, a flavin-containing PAS domain, or disulfide bond formation (Table 3).

**TABLE 3:** Sensors for oxygen and redox potential in c-di-GMP networks

Bacterial species	Protein	Molecule^*a*^	Sensory domain and cofactor or mechanism	Function	Evidence^*b*^	Reference(s)
Acetobacter xylinum	*Ax*PDEA1	O_2_ (−)	PAS9, heme	PDE	IDA	51
Escherichia coli	PdeO	O_2_ (+), CO (+)	PAS9, heme	PDE	IDA	50, 167, 226–229
Escherichia coli	DgcO	O_2_ (+), CO (+), HCN (+), redox	Protoglobin, heme	DGC	IDA	76 – 81
Vibrio cholerae	*Vc*Bhr-DGC	O_2_ (−)	Hemerythrin, Fe^2+^/^3+^	DGC	IDA	71
*Ferrovum* sp. PN-J185	Bhr-HD-GYP	O_2_ (−), redox	Hemerythrin, Fe^2+^/^3+^	PDE	IDA	73
Azorhizobium caulinodans ORS571	Chp1	O_2_ (+)	Protoglobin, heme	PDE	IMP, IDA	230
Bordetella pertussis	*Bpe*GReg	O_2_ (+)	Protoglobin, heme	DGC	IDA	81, 231–234
Azotobacter vinelandii	*Av*GReg	O_2_ (+)	Protoglobin, heme	DGC	IDA	233, 235
Pectobacterium carotovorum subsp. *carotovorum*	PccGCS	O_2_ (−)	Protoglobin, heme	DGC	IDA	232, 236
Pseudomonas aeruginosa	RbdA	O_2_ (+)	PAS1, heme	PDE	IMP	237
Shewanella putrefaciens CN32	DosD	O_2_ (+)	Protoglobin, heme	DGC	IMP, IDA	238
Pseudomonas aeruginosa	RmcA	Phenazines (+); redox (+ oxidized), O_2_ (+) O_2_ (+)	PAS3; PAS9, FAD	PDE	IMP	48, 172
*Acetobacter xylinum*	*Ax*DGC2	Redox (+ oxidized)	PAS9, FAD	DGC	IDA	239
Yersinia pestis	HmsC	Redox (+ reduced)	Disulfide reduction	regulator	IDA	178, 240
	HmsD			DGC	IMP	178, 240
Bacillus cereus	CdgF	Redox (+DGC oxidized, +PDE reduced)	PAS9, flavin	DGC-PDE	IMP, IDA	176
Escherichia coli	PdeC	Redox (+ reduced)	CSS, disulfide reduction	PDE	IMP, IDA	101

a The (+) denotes an activator of the protein, whereas the (−) denotes an inhibitor.

b Categorized using gene ontology (GO) evidence codes are as follows: IDA, inferred from direct assay; IMP, inferred from mutant phenotype.

Two of the best-studied examples of O_2_-sensing proteins are DgcO and PdeO, which were first identified in E. coli (167). DgcO coordinates heme via its protoglobin domain (e.g., globin-coupled sensor). In the purified DgcO protein, O_2_ binds to the heme, and this binding is stabilized by aromatic amino acids that are distal to the cysteine residue that serves as the covalent heme attachment site (75, 76, 78, 79). O_2_ binding activates the DgcO GGDEF domain, increasing c-di-GMP synthesis. In contrast, PdeO coordinates heme via a PAS9 domain (i.e., a domain that belongs to the same Pfam domain family as LOV but is not an LOV domain) (75, 167). O_2_ binding to the heme cofactor in PdeO stimulates its EAL domain, leading to c-di-GMP degradation. The transcription of the mRNAs for these proteins is coupled in the *dgcO-pdeO* operon (previously named *yddV*-*yddU* and the *dosCP* operon). DgcO and PdeO assemble into a functional complex *in vitro* and, under certain conditions (e.g., in stationary-phase cultures), are also thought to comprise part of the E. coli RNA degradosome via interactions with a scaffolding protein, RNase E (168, 169). Another component of the degradosome is polynucleotide phosphorylase (PNPase), which is a c-di-GMP response enzyme in RNA metabolism that serves as a 3′ polyribonucleotide polymerase or a 3′-to-5′ exoribonuclease (168). Together, DgcO and PdeO function within this macromolecular complex—termed the oxydegradosome—to fine-tune c-di-GMP levels that drive O_2_-dependent RNA processing via PNPase (168).

A recent study characterized the role of a hemerythrin domain in controlling PDE activity in Bhr-HD-GYP, a protein from the iron-oxidizing bacterium *Ferrovum* sp. strain PN-J185 (Table 3). This organism was isolated from an acid mine drainage site and can grow at pH values below 4. In such sites, bacteria can take advantage of the stability of ferrous iron at low pH and use it as an electron donor for growth by aerobic respiration; in neutral-pH environments, ferrous iron is not typically available as an electron donor because it reacts rapidly with O_2_. Bhr-HD-GYP contains an N-terminal hemerythrin domain and a C-terminal HD-GYP domain. Purified Bhr-HD-GYP was shown to bind iron only in the reduced (ferrous) form and not the oxidized (ferric) form. These two states were reversibly interconverted in the purified protein, suggesting that the domain is used for redox sensing. Although exposure to air oxidized the protein, absorption spectra did not show evidence of O_2_ binding, indicating that an O_2_ adduct does not form (in contrast to what is observed for invertebrate hemerythrins) (73, 170). In the oxidized state, no PDE activity was detected. However, in the reduced state, a time-dependent decrease in c-di-GMP concentration was measured, which suggests that the reduced state is the active form of the PDE protein (73). The role of secondary messaging in *Ferrovum* sp. PN-J185 is not known, but the ability to make c-di-GMP-dependent lifestyle changes in response to redox state may contribute to bacterial survival in the harsh acid mine drainage environment.

Redox state can influence protein conformation via PAS domains when redox-active small molecules or cofactors bind to these sensory domains. In addition to conferring a light-dependent phenotype (described above), the P. aeruginosa protein RmcA has also been implicated in the response of P. aeruginosa to its own redox-active products, a class of small molecules called phenazines. These compounds contribute to redox homeostasis by coupling the oxidation of NADH to the reduction of O_2_. Accordingly, a phenazine-null mutant shows an increased NADH/NAD^+^ ratio (171). Like the effect of light on P. aeruginosa biofilm development, the production of phenazines by the wild type inhibits wrinkling (i.e., a phenazine-null mutant forms wrinkles earlier than its phenazine-producing parent) (172). RmcA is required for this effect (48). RmcA PASd (the fourth PAS domain from the N terminus) is similar to flavin-binding LOV domains and is required for the light-dependent effects on biofilm development. PASd is also required for phenazine-dependent inhibition of wrinkling, suggesting that phenazines may affect the redox state of the bound flavin. One possible mechanism is that phenazines directly, or indirectly via the NADH/NAD^+^ ratio, modulate the redox state of the flavin in the RmcA PASd domain. Alternatively, phenazines, which bear some similarity to the flavin isoalloxazine ring in that they are composed of three fused heterocycles, may bind one of the PAS domains in RmcA. Intrinsic fluorescence quenching experiments indicated that phenazines bind to RmcA, and computational modeling and genetic approaches have provided some clues as to the cofactors that may bind to each of the four PAS domains of P. aeruginosa RmcA (48). Interestingly, the PASa-c domains of RmcA are predicted to bind different cofactors. PASa (the most N-terminal PAS domain), which appears to control a condition-dependent DGC activity of RmcA, is predicted to bind phenazines; PASb is predicted to bind a lipid molecule; and PASc is predicted to bind heme (173). The unique features of each of the RmcA PAS domains suggest that this protein integrates a broad spectrum of stimuli to influence multicellular outputs (Fig. 3).

Although c-di-GMP-dependent signaling pathways have been elucidated in greater detail in Gram-negative bacteria, they also function in Gram-positive bacteria (174). The Bacillus cereus group is a cohort of *Bacillus* strains that are closely related phylogenetically (i.e., they have similar 16S sequences) but that have been historically treated as separate species due to other differences, such as the presence of unique plasmids (175). Members of this group have been studied due to their production of toxins, their pathogenicity, and their applications in agriculture. Ten proteins with the potential to modulate c-di-GMP levels are conserved among members of the Bacillus cereus group. In a study characterizing the physiological roles of these proteins, the protein CdgF stood out due to the particularly strong effects of *cdgF* overexpression or deletion on c-di-GMP levels, biofilm formation, and motility (176).

CdgF has an N-terminal PAS9 domain and tandem GGDEF and EAL domains with intact catalytic sites, but mutant analyses indicated that it functions primarily as a DGC *in vivo*. The UV-visible (UV-vis) spectrum of recombinant, purified CdgF suggests that it binds a flavin molecule. Shifting the protein from the oxidized to the reduced state correlated with a switch from DGC to PDE activity. The authors of this study thus speculated that CdgF acts to promote biofilm formation in oxygenated environments (176), and this idea is consistent with the noted ability of B. cereus group members to form pellicles, which are biofilms at air-liquid interfaces.

Diverse Gram-negative bacteria have protein systems that sense the redox state of the periplasm and transduce this information into modulation of c-di-GMP levels in the cytoplasm. Compared with the conditions of the cytoplasm, those of the periplasm are more sensitive to the external environment. While the cytoplasm is typically maintained in a reducing state, the periplasm is oxidizing and supports the generation of disulfide bonds, which form between two cysteines in proteins or small molecules. Periplasmic disulfide bond formation can be an important component of protein folding or regulation of activity and is usually mediated by well-characterized Dsb proteins, which facilitate electron transfer between target proteins/small molecules and the quinone pool of the electron transport chain (177). For several protein systems from bacteria such as Yersinia pestis, Escherichia coli, Salmonella enterica serovar Typhimurium, and Pseudomonas aeruginosa, genetic and biochemical approaches have been used to show that disrupting the function of the Dsb proteins or adding chemicals that alter the redox conditions of the periplasm affects c-di-GMP synthesis and/or degradation. The proteins catalyzing these activities generally have N-terminal periplasmic domains that (i) interact with redox-sensitive extracytoplasmic proteins or (ii) themselves contain a redox-sensitive cysteine pair that forms a disulfide bond.

Y. pestis is the causative agent of plague and is carried by fleas. The formation of robust biofilms in the flea gut promotes the transmission of Y. pestis to mammalian hosts. Biofilm formation is stimulated by reducing conditions and is positively regulated by c-di-GMP. A recent study implicated the HmsCD proteins in this effect (178). HmsC is a periplasmic protein that contains multiple cysteine pairs capable of forming disulfide bonds, while HmsD is a DGC that contains an N-terminal periplasmic domain and a cytoplasmic GGDEF domain. The authors found that the redox conditions of the periplasm affect HmsC abundance, with oxidizing conditions corresponding to high levels of HmsC and reducing conditions corresponding to low levels of this protein. Furthermore, HmsC was found associated with HmsD specifically under oxidizing conditions. These observations support a model in which the oxidized form of HmsC binds to HmsD and acts to inhibit c-di-GMP production and therefore biofilm formation.

E. coli PdeC is an example of a transmembrane c-di-GMP-modulating protein that directly responds to the redox state of the periplasm via an N-terminal domain that forms a disulfide bond. This N-terminal region contains two TM segments with an intervening periplasmic loop referred to as a CSS-motif domain. This loop has two well-conserved cysteine residues, of which one is found in a characteristic CSS motif (102). The cytoplasmic portion of the protein contains an EAL domain, which confers PDE activity. Herbst et al. found that the expression of wild-type PdeC inhibits biofilm matrix production in E. coli (101), which is consistent with PdeC degrading c-di-GMP *in vivo*. Genetically eliminating one or both cysteines in the CSS domain further inhibited matrix production, indicating that disulfide bond formation between these residues inhibited the PDE activity of PdeC. Through a series of genetic and biochemical experiments, the authors showed three possible states for PdeC: (i) one in which PdeC forms the disulfide bond in the CSS domain, which results in low PDE activity and therefore relatively high concentrations of c-di-GMP; (ii) one in which the cysteines in the CSS domain remain in the free thiol form, allowing them to be used for dimerization with another PdeC molecule, which increases PDE activity and therefore decreases c-di-GMP concentration; and (iii) one in which the region that contains the free thiols is processed by periplasmic proteases to yield a shorter protein that is still able to dimerize and degrade c-di-GMP and therefore decrease the c-di-GMP concentration. The formation of the first state is possible when cells are grown under aerobic conditions. In this case, the DsbA/DsbB system and the oxidizing environment of the periplasm promote disulfide bond formation. The second and third states occur in reducing environments, which favor the formation of free thiols as opposed to disulfide bonds. After the third state has been formed, it is slowly degraded by further proteolysis. Together with *in vivo* results, these observations indicate that PdeC functions to modulate matrix production in poorly oxygenated subzones of E. coli biofilms. The fact that PdeC is just one of five similar CSS-domain PDEs in E. coli suggests that periplasmic conditions exert a substantial influence on c-di-GMP-dependent processes in this organism and exemplify the potential for elaborate linkages between environmental sensing and multicellular behavior.

### Thermal sensing.

Temperature affects c-di-GMP levels in a variety of bacterial species (5, 179–182), and yet the mechanisms of thermal sensing remain poorly understood. For example, the diguanylate cyclase HmsT from Yersinia pestis (179), which is comprised of a solo GGDEF domain with no identifiable sensory domain, is thought to be regulated by temperature at translational and posttranslational levels. In this case, Y. pestis displays elevated levels of c-di-GMP at 21°C relative to 37°C, and a loss of the 3′ untranslated region (UTR) of *hmsT* mRNA abolishes this phenotype (183, 184). Also, HmsT is degraded at 37°C by Lon protease, presumably through exposure of one or more cleavage sites in HmsT at that temperature (185).

**FIG 5:**
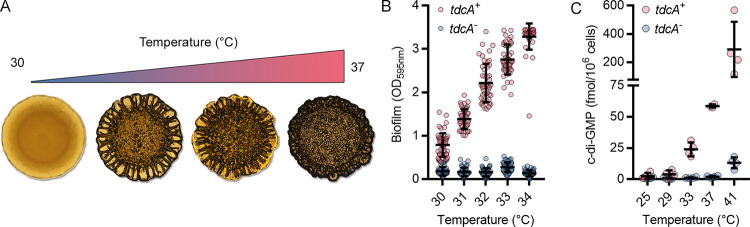
The thermosensory diguanylate cyclase fine tunes Pseudomonas aeruginosa biofilm formation and c-di-GMP levels in response to temperature. (A) Colony morphology of P. aeruginosa CF39S (*tdcA*^+^) at 30, 32, 34, and 37°C (from left to right). (B) Temperature-dependent biofilm formation by P. aeruginosa CF39 (*tdcA*^−^) and CF39S (*tdcA*^+^). (C) Temperature-dependent increases in intracellular c-di-GMP from P. aeruginosa CF39 (*tdcA*^−^) and CF39S (*tdcA*^+^). Data were taken from reference 5.

Almblad, Randall, and colleagues (5) have recently discovered a thermosensory diguanylate cyclase in P. aeruginosa (TdcA_Ps_). Functional homologs of TdcA were also identified in distantly related proteobacteria (5). TdcA_Ps_ synthesizes c-di-GMP with catalytic rates that increase >140-fold over a 10°C change (Fig. 5). Although the underlying biochemistry is different, these high enzymatic rate-temperature dependencies are analogous to those described for the thermosensitive-transient receptor potential (thermoTRP) proteins, which are the hot or cold-sensing proteins of neurons (186–188). The thermal-sensing functions of TdcA are linked to a thermosensitive Per-Arnt-SIM (thermoPAS) domain. This conclusion was evidenced by the design of chimeric proteins in which the thermoPAS domain was spliced to other effector domains to produce designer enzymes with thermosensitive functions (5). While this was the first description of thermal sensing for the widespread family of PAS protein domains and the biophysical mechanism of thermal sensing remains unknown, the authors demonstrated that TdcA mediates c-di-GMP-dependent thermotransduction that regulates biofilm formation, motility, and virulence in P. aeruginosa (5).

### Chemosensing.

c-di-GMP networks sense diverse chemicals (Table 4 and 5), which may originate on the inside or outside cells. In the sections below, we summarize known c-di-GMP-dependent chemosensory mechanisms for nitric oxide (NO) (Table 4). Subsequently, we discuss chemosensory mechanisms for cellular nucleotides, amino acids and sugars, quorum-sensing molecules, and other biotic and abiotic compounds (Table 5).

**TABLE 4:** Nitric oxide (NO) sensors in c-di-GMP networks

Bacterial species	Protein	Molecule^*a*^	Sensory domain and cofactor	Function	Evidence^*b*^	Reference(s)
Legionella pneumophila	Hnox1	NO (−)	HNOB, heme	Sensor	IDA	90
	Lpg1057			DGC	IDA	90
Dinoroseobacter shibae DFL12	H-NOX	NO (−)	HNOB, heme	Sensor	IMP, IDA	190
	Dgc1			DGC	IMP, IDA	190
Shewanella woodyi	SwH-NOX	NO (−)	HNOB, heme	Sensor	IMP, IDA	91
	SwDGC			DGC-PDE	IDA	91
Shewanella oneidensis	HnoX	NO (−)	HNOB, heme	Sensor	IDA	83, 89
	HnoK			Kinase	IDA	83
	HnoB		RRRD	PDE	IDA	83
Legionella pneumophila	NosP	NO (−DGC, +PDE)	FIST-FIST_C	Sensor	IMP, IDA	113, 114
	NahK			Kinase	IDA	113
	NarR		RRRD	DGC-PDE	IDA	113

a The (+) denotes an activator of the protein, whereas the (−) denotes an inhibitor.

b Categorized using gene ontology (GO) evidence codes as follows: IDA, inferred from direct assay; IMP, inferred from mutant phenotype; IEP, inferred from expression pattern.

**TABLE 5:** Other chemoreceptors in c-di-GMP networks

Molecule(s)^*a*^	Bacterial species	Protein	Sensory domain	Function	Evidence^*b*^	Reference(s)
Nucleotides						
GTP (+)	Pseudomonas aeruginosa	FimX	Degenerate GGDEF	PDE	IDA, IMP	191
cNMP (cAMP/cGMP) (+)	Bdellovibrio bacteriovorus	Bd1971	Cyclic nucleotide-binding domain	PDE	IDA	6
cAMP (+)	Leptospira interrogans	Lcd1	GAF1	DGC	IDA	193
cAMP (+)	*Thermosynechococcus vulcanus*	Tlr0485	GAF1	PDE	IDA	194
GDP (−)	Mycobacterium smegmatis	DcpA	GAF2	DGC	IDA	195
Amino acids and sugars						
Glucose (−), *N*-acetyl- glucosamine (−), maltose (+), mannose (+), fructose (+)	Vibrio cholerae	EIIA^Glc^	PTS_EIIA_1	Regulator	IMP, IDA	111
		PdeS		PDE	IMP, IDA	111
Proline (−), valine (−), isoleucine (−)	Aeromonas veronii	SpdE	dCACHE1	DGC	IMP, IDA	197
l-Arginine (+)	Pseudomonas aeruginosa	RmcA	SBP_bac_3	PDE	IDA, IEP	34
l-Arginine (+)	Salmonella Typhimurium	STM1987	dCACHE1	DGC	IMP, IDA	198
Quorum-sensing molecules						
Autoinducer-2 (AI-2) (+)	Rhodopseudomonas palustris	rpHK1S-Z16	dCACHE1	DGC	IDA	9
*cis*-2-Dodecenoic acid (BDSF) (+)	Burkholderia cenocepacia	RpfR	PAS9	PDE	IMP, IDA	10, 11
Others						
Citrate (+)	Pseudomonas fluorescens	GcbC	dCACHE1	DGC	IMP	35
Ethanol (+)	Pseudomonas aeruginosa	Wsp apparatus	4HB_MCP_1	sensor	IMP, IDA	7
Heme (−)	Vibrio cholerae	CdpA	FIST-FIST_C	PDE	IDA	115
Zinc (Zn^2+^) (−), hocl (+)	Escherichia coli	DgcZ (YdeH)	CZB	DGC	IMP, IDA	69, 70

a The (+) denotes an activator of the protein, whereas the (−) denotes an inhibitor.

b Categorized using gene ontology (GO) evidence codes as follows: IDA, inferred from direct assay; IEP, inferred from expression pattern; IMP, inferred from mutant phenotype.

### Nitric oxide.

Compelling biochemical and genetic evidence indicates that NO sensing can be carried out by auxiliary proteins of TCSs containing the HNOB domain and perhaps the FIST-FIST_C domain as well (Table 4). One key example is the HnoX-dependent pathway that is found in Shewanella oneidensis and Vibrio cholerae (Table 4) (189). HnoX, which contains an HNOB domain (83), is an auxiliary protein to the HnoKB TCS that interacts with the sensor kinase HnoK (83, 89). In this case, NO promotes biofilm formation. In the absence of NO, HnoX activates the HnoK autokinase, which phosphorylates the RRRD domain of the PDE HnoB, leading to degradation of c-di-GMP and inhibition of biofilm formation (83, 89). When NO is present, HnoX inhibits HnoK thereby reducing HnoB phosphorylation and PDE activity and promoting biofilm formation (83). The activity of HnoB is also fine-tuned by another protein, HnoD, which is an inhibitor of HnoB (83).

An analogous TCS is the NosP-NahK-NarR system, but in contrast to HnoXKBD system, NO promotes PDE activity (Fig. 3). Here, NosP binds to NO, which might occur via its solo FIST-FIST_C domain. NO-bound NosP activates NahK autokinase activity, which phosphorylates the RRRD domain of NarR, activating the NarR EAL domain (Fig. 3). In the absence of NO, NarR remains unphosphorylated and displays enhanced DGC activity via its GGDEF domain (113, 114).

A conspicuous trend that emerges from this review is that NO often modulates the activities of DGCs and c-di-GMP-specific PDEs via an effector protein (Table 4). There are many examples. For instance, the Dgc1 diguanylate cyclase from Dinoroseobacter shibae is inhibited by an HNOB domain-containing partner protein called H-NOX when it binds NO (190). Similarly, the DGC Lpg1057 from Legionella pneumophila is inhibited by the HNOB domain-containing protein Hnox1 when that partner protein binds NO (90). Moreover, *Sw*DGC from Shewanella woodyi, which contains functional EAL and GGDEF domains, binds to a partner protein, *Sw*H-NOX, that is required for its activity (91). In the absence of NO, *Sw*DGC displays increased DGC activity but minimal PDE activity, while in the presence of NO, this protein complex displays minimal DGC activity and increased PDE activity (91).

### Nucleotides.

Several OCSs in c-di-GMP networks are known to sense GDP, GTP, and cyclic nucleotide monophosphates (cNMPs) (Table 5). One mechanism of sensing relies on a degenerate GGDEF domain, which has been observed in the EAL domain-containing FimX protein from P. aeruginosa (191). The PDE activity of Fimis increased by GTP but only when the degenerate GGDEF motif (GDSIF) remains intact (191). Another documented mechanism of nucleotide sensing involves the cNMP_binding domain, which binds both 3′,5′-cyclic AMP (cAMP) and 3′,5′-cyclic GMP (cGMP) and has been observed in the EAL domain-containing protein Bd1971 from Bdellovibrio bacteriovorus (6). In *Bdellovibrio*, c-di-GMP regulates both its predatory or axenic life cycle, as well as swimming and swarming behaviors (192). The Bd1971 protein hydrolyzes c-di-GMP only in the presence of cAMP and cGMP (6). *Bdellovibrio* contains many annotated adenylate cyclases (6) that provide intricate controls over cAMP production throughout its life cycle. Thus, Bd1971 exemplifies additional intricacies in these signaling networks that arise from cross talk between second messengers that enable complex cellular decision-making.

Another group of proteins relies on GAF domains to detect cAMP (Table 5). For example, cAMP induces a conformational change in the GAF1 domain of the Lcd1 protein from Leptospira interrogans, activating the adjacent GGDEF domain and c-di-GMP synthesis (193). An analogous mechanism might operate for the cAMP-responsive Tlr0485 protein from Thermosynechococcus vulcanus in which a GAF1 domain is linked to an HD-GYP domain (194). Lastly, the binding of GDP to the GAF2 domain of the DcpA DGC from Mycobacterium smegmatis is thought to lead to conformation changes that lead to the inhibition of c-di-GMP production by the connected GGDEF domain (195). Taken together, these examples not only provide clues about mechanisms of cross talk between second messengers but also make it tempting to speculate that some c-di-GMP signaling proteins may be wired to respond to cellular energy state.

### Aminto acids and sugars.

Diverse environmental nutrients stimulate c-di-GMP signaling networks, consistent with well-known nutrition-dependent phenotypes for biofilm formation and swarming motility, which are coordinated by c-di-GMP signaling.

Emerging evidence from Vibrio cholerae links carbohydrates to the regulation of c-di-GMP metabolism via protein EIIA^Glc^, which modulates the activity of a c-di-GMP-specific PDE, PdeS, as part of an oligomeric complex (111). During carbon starvation (111) or exposure to various carbon sources (including maltose, mannose, and fructose) (196), the PTS_EIIA_1 domain of EIIA^Glc^ is phosphorylated by the PTS, activating PdeS PDE activity through quaternary interactions. In contrast, *N*-acetylglucosamine and glucose (196) promote EIIA^Glc^ dephosphorylation through phosphate transfer to these sugars, inhibiting PdeS (110).

Another recent discovery exemplifies amino acid sensing. SpdE from Aeromonas veronii is a DGC that uses a dCACHE_1 domain to sense proline, valine, and isoleucine (197). Structural analysis revealed that the amino acid ligands bind to the dCACHE_1 domain via a series of critical amino acid residues in a binding pocket that can fit only small hydrophobic amino acids. Proline, valine, and isoleucine inhibit SpdE DGC activity, directly increasing the speed of cells, as measured by microscopy. This process, called chemokinesis, works in concert with chemotaxis to facilitate the rapid movement of *A. veronii* into the host in the presence of SpdE-inhibiting ligands (197).

Work by Mills et al. used a fluorescence resonance energy transfer (FRET)-based c-di-GMP biosensor (198, 199) in conjunction with flow cytometry of live cells to screen a chemical library of environmental nutrients that might modulate intracellular c-di-GMP in *S.* Typhimurium. Although cells in the population displayed heterogeneity in their response to the stimuli, the authors identified that within subpopulations of cells, many compounds can induce increases in c-di-GMP levels (e.g., adenosine, glucose, l-arginine, l-lysine, phytic acid, and *N*-acetylglucosamine), while others decrease them significantly (e.g., d-glutamate, m-hydroxyphenylacetic acid [mHPAA], salicylic acid, butyric acid, and l-pyroglutamate). Moreover, micromolar concentrations of these compounds caused corresponding changes in cellulose levels. l-Arginine was exceptional among the identified environmental nutrients because *S.* Typhimurium cells responded to it at very low concentrations (e.g., 0.128 to 0.64 μM). Genetic linkage analysis identified that a Δ*stm1987* mutant lost its ability to respond to l-arginine, although the authors noted three other genes that might encode secondary, downstream c-di-GMP-metabolizing enzymes that might contribute to the phenotype, albeit to a lesser extent than *stm1987* (198). Genetic methods were further used to identify that the periplasmic dCACHE_1 domain of STM1987 was required for l-arginine sensing, as was the putative periplasmic l-arginine-binding protein ArtI (198).

### Quorum-sensing molecules.

Because quorum sensing regulates a constellation of genes (200, 201), dissecting regulatory relationships between it and c-di-GMP has not been straightforward. For instance, genetic and transcriptomic analyses have revealed complicated, QS-dependent transcriptional regulation for genes involved in the control of c-di-GMP (202), of which some may additionally drive posttranslational modification of DGCs (12). Nevertheless, there is direct evidence for at least two receptors for quorum-sensing molecules in c-di-GMP networks.

First, the diffusible signal factors, which are fatty acids that share in common a *cis*-2 double bond, are an important group of quorum-sensing autoinducers. Structural biology has provided evidence indicating that the *Burkholderia* diffusible signaling factor (BDSF) interacts with the PAS9 domain of the c-di-GMP specific PDE RpfR (10). Upon the binding of BDSF to its PAS9 domain, RpfR displays enhanced PDE activity, leading to reduced cellular c-di-GMP levels and biofilm dispersion (10, 11). While this discovery was made in Burkholderia cenocepacia (11), the BDSF-RpfR quorum-sensing system appears widespread in *Betaproteobacteria* and *Gammaproteobacteria* (203).

Second, Zhang and colleagues recently identified a group of dCACHE domains that function as receptors that preferentially bind boron-free autoinducer 2 (AI-2). Membrane-associated DGC rpHK1S-Z16 from Rhodopseudomonas palustris interacts with AI-2 through its dCACHE_1 domain, as evidenced by isothermal titration calorimetry (9). Also, membrane fractions containing rpHK1S-Z16 have enhanced DGC activity in the presence of AI-2 (9). Nevertheless, the authors executed all this work *in vitro*, and so the physiological function for AI-2 binding of rpHK1S-Z16 in R. palustris remains to be determined.

### Other biotic and abiotic molecules.

Accumulating studies reveal additional, diverse chemosensory functions for c-di-GMP networks, including the sensing of tricarboxylic acid intermediates, host factors, and metal ions. For instance, Pseudomonas fluorescens GcbC likely binds to citrate via its dCACHE_1 domain, as evidenced by a homology model of the dCACHE_1 domain, which was based on a crystal structure from rpHK1S-Z16 from R. palustris, a homolog of GcbC. A mutational analysis was used to confirm that the critical residues for binding citrate in rpHK1S-Z16 were operational in citrate sensing for P. fluorescens GcbC (35). Further research indicated that GcbC binds directly to the inner-membrane effector protein LapD, which, after binding c-di-GMP, leads to enhanced biofilm formation (35). When GcbC is interacting with LapD through a direct protein-protein interaction (204), citrate is able to enhance DGC activity (35). However, in the absence of LapD, citrate has no effect on GcbC activity (35).

In V. cholerae, CdpA may rely on FIST-FIST_C domains to detect heme. Heme binding inhibits the EAL domain of CpdA (115), and therefore, heme leads to increased intracellular c-di-GMP levels in V. cholerae.

Finally, numerous studies have linked bacterial exposure to metal or metalloid ions to modulation of c-di-GMP levels (205–208). Mechanistic insight comes from investigating E. coli DgcZ, which responds to femtomolar concentrations of Zn^2+^ via its CZB domain (69, 70). Increasing Zn^2+^ concentrations *in vitro* led to the strong inhibition of DGC activity (69, 70). Interestingly, the oxidation of the critical cysteine residue in the CZB domain of DgcZ by hypochlorite allows for increased DGC activity in the presence of Zn^2+^, indicating that cellular oxidants, which are also affected by metal toxicity (209, 210), can modulate DgcZ activity too (13).

## USING THE SENSES: ENGINEERING c-di-GMP NETWORKS FOR BIOTECHNOLOGY

An understanding of sensory perception in c-di-GMP networks is providing an opportunity to use these systems for biotechnology. The most notable application so far has been optogenetics, which relies on the heterologous expression of genes coding for light-sensitive proteins and illumination to alter cellular behavior (211). Light is an easily controllable, noninvasive factor that can be used to regulate photosensitive DGCs and PDEs with high spatial and temporal precision (212). One example is BphS, a synthetic DGC that contains a sensory phytochrome domain from the Rhodobacter sphaeroides BphG protein (Table 1) and the GGDEF domain from the *Synechocystis* spp. Slr1143 protein (213). BphS responds to near-infrared light that can penetrate deep into animal tissues, which is highly advantageous for *in vivo* experiments (213). BphS can also be coupled with a blue light-responsive PDE—such as EB1, a synthetic protein made from the PDE BldP (212), or BlrP1, the naturally occurring K. pneumoniae protein (Table 1)—to create a dichromatic optogenetic module where c-di-GMP levels can be increased or decreased (212, 214). This dual control system is beneficial when studying c-di-GMP signaling at short time scales and can also be used for bioprinting engineered biofilms (214, 215). The flavin cofactor is readily available across many species, and thus, optogenetic tools using LOV or BLUF domains are a robust choice for synthetic sensors (216).

## CONCLUSIONS AND OUTLOOK

To date, sensory perception in c-di-GMP networks has been studied in at least 40 bacterial species from 35 genera (Tables 1 to 5; see Table S3 in the supplemental material). Investigations of sensory perception in these networks, therefore, broadly advance knowledge of how bacteria modulate their physiology, community structure, and behaviors in response to physical and chemical gradients or fluxes in very diverse environments.

Despite recent advances, we propose that most sensory functions of c-di-GMP networks remain undiscovered. This idea is evidenced by the diverse repertoire of putative modular sensory and receiver domains predicted to be in c-di-GMP signaling proteins (Fig. 4; Tables S1 and S2). Many of these putative sensor domains have no ascribed function, and moreover, it is likely that many sensor domains cannot yet be detected via bioinformatics at all. Also, despite evidence and predictions that c-di-GMP networks may sense antibiotics (217) or lipids (173), for example, a sensor domain for these molecules has yet to be identified in these systems.

Many gaps in our knowledge of well-studied sensor systems remain too. For example, the molecular basis for single-cell heterogeneity in c-di-GMP-dependent chemosensing (198) or surface sensing (218) is not understood. Such observations might provide fresh insight into the division of labor in bacterial populations (218). Also, the literature is rife with descriptions of genetic linkages between histidine kinases and stimuli of c-di-GMP networks (Table S3); however, direct demonstrations of interactions between stimuli and sensory domains of histidine kinases remain exceptionally rare for c-di-GMP networks. Considering the large number of putative RRRD (or REC) domains in proteins with GGDEF, EAL, and/or HD-GYP domains (Fig. 4; Tables S1 and S2), this knowledge gap in c-di-GMP signaling is significant. We also posit that conclusions based solely on genetic linkage analyses might have led to the premature assignment of sensory functions to some TCSs, especially if diverse stimuli have been linked to the same gene in multiple studies (Table S3).

Finally, investigations of sensory perception in c-di-GMP networks are leading to innovations in optogenetics and may provide other biosensors for use in synthetic biology. Such modular sensory devices could be used to sense temperature, O_2_, and metal ions and to control bioprocesses in a variety of research and industrial contexts. It is also possible that sensory perception may be exploited to devise new therapeutics to treat infections with biofilm etiology.
